# Proteomics and Food Analysis: Principles, Techniques, and Applications

**DOI:** 10.3390/foods10112538

**Published:** 2021-10-22

**Authors:** Mónica Carrera

**Affiliations:** Food Technology Department, Institute of Marine Research (IIM), Spanish National Research Council (CSIC), 36208 Vigo, Pontevedra, Spain; mcarrera@iim.csic.es; Tel.: +34-986231930

Proteomics can be considered the discipline of the large-scale analysis of proteins in a particular biological system. Proteomics comprises the study of the structure and function of proteins, the quantification of protein abundance, the determination of protein intracellular location, the analysis of protein modifications and the study of protein–protein interaction networks. Mass spectrometry (MS)-based proteomics has been recognized as an indispensable tool to precisely identify and quantify thousands of proteins from complex protein samples, and is used in the majority of proteomics studies. Moreover, bioinformatics treatment of MS data has increased the scale of proteomics tools, representing a powerful strategy for high-throughput protein and peptide identification and quantification. In this regard, two sequential food proteomics approaches (discovery food proteomics and targeted food proteomics) are the main proteomic tools used for food quality and safety studies. The aim of discovery food proteomics is to analyze a particular proteome to identify potential food protein/peptide biomarkers, commonly using a bottom-up proteomics strategy in which the proteins of interest are digested into peptides using proteases (i.e., trypsin), and the resulting peptides are analyzed by MS. Then, targeted food proteomics methods are used to search for the peptide biomarkers selected in the discovery phase in biological samples or in food products with high precision, sensitivity and reproducibility. Thus, proteomics methodologies are an advantageous strategy for food science studies, where research institutions, agencies, food industries, and regulatory laboratories are combining efforts to acquire necessary knowledge on food composition, quality, and safety. The potential of proteomics in food analysis is highlighted in this special issue on different subjects concerning food quality and safety. The six scientific papers that contribute to this Special Issue, “Proteomics and Food Analysis: Principles, Techniques, and Applications”, provide an excellent overview of the wide-ranging proteomics approaches applied to food analysis.

In this context, the manuscript published by Daher et al., “Sensopeptidomic kinetic approach combined to decision trees and random forest to study the bitterness during enzymatic hydrolysis kinetics of micellar caseins”, reports the proteomic characterization of protein hydrolysates responsible for the unpleasant taste in milk [1]. Milk protein hydrolysates have significant advantages in sport nutrition and elderly and infant nutrition, as the use of these hydrolysates induces a very rapid release of amino acids in the blood, which maximizes muscle protein anabolism and facilitates body recovery and nutrition. However, hydrolyzed proteins can sometimes have an unpleasant bitter taste or off flavors, which limits the breadth of their applications in nutrition. In this article, the authors performed an interesting investigation of peptide characterization of micellar caseins using proteolytic enzymes and liquid chromatography-electrospray-quadrupole-time-of-flight-tandem mass spectrometry (LC-ESI-Q-TOF-MS/MS) analysis. Moreover, they correlated the amino acid structure of the identified peptides with the bitterness properties of micellar casein hydrolysates by using different statistical and bioinformatics tools based on differential expression analysis, heat maps, regression trees and random forests. The authors concluded that the formulated hydrolysates may be used in the development of future food formulations such as new peptide-fortified ready-to-drink infant formulas.

An interesting proteomics approach reported by Abril et al. in “Proteomic characterization of bacteriophage peptides from the mastitis producer *Staphylococcus aureus* by LC-ESI-MS/MS and bacteriophage phylogenomic analysis” describes the characterization of bacteriophage peptides from the mastitis-causing bacterium *Staphylococcus aureus* (*S. aureus*) isolated from dairy products [2]. *S. aureus* is considered one of the major foodborne pathogens that can cause serious food intoxication in humans due to its production of endotoxins. This bacterium remains a major problem in the dairy industry due to its persistence in cows, its pathogenicity, its contagiousness and its ability to easily colonize the skin and mucosal epithelia. The authors used a shotgun proteomics approach (in a gel-free strategy where a complex mixture of food proteins is digested in solution with a protease such as trypsin, and the resulting mixture of peptides is then analyzed by LC-MS/MS) for the characterization of 20 different *S. aureus* strains. For this purpose, they utilized an LC-ESI-MS/MS-based workflow using an LTQ-Orbitrap instrument to identify relevant phage-specific peptides of several *S. aureus* strains to identify both phages and bacterial strains. Additionally, phylogenomic trees were developed to demonstrate a link between phage phylogeny and their ability to infect the same bacterial species. The authors concluded based on the data obtained for the different models of mastitis that the phage therapy using bacteriophages in this study may be considered an innovative alternative to antibiotics for the treatment of mastitis caused by *S. aureus*.

The manuscript published by Monaci et al., “Validation of a MS-based proteomics method for milk and egg quantification in cookies at the lowest VITAL levels: an alternative to the use of precautionary labeling”, presents for the first time the development of a targeted proteomics approach based on the multiple reaction monitoring (MRM) method on a triple quadrupole MS instrument for milk and egg identification and quantification in processed foods such as cookies [3]. The method allows the detection of milk and egg proteins at levels lower than the 0.2 mg recommended by the Voluntary Incidental Trace Allergen Labeling (VITAL) program as necessary to help food producers avoid cross-contamination. As stated by the authors, this could be used as an alternative method for precautionary allergen labeling, alongside other common detection methodologies based on proteins or nucleic acids. The authors conclude that this targeted proteomics method could represent, a promising tool to be implemented along the food chain to detect even tiny amounts of allergens contaminating food commodities.

In the article written by Ham et al., “Increasing coverage of proteome identification of the fruiting body of *Agaricus bisporus* by shotgun proteomics”, the authors provide an overview on the protein identification of the fruit body of *Agaricus bisporus*, analyzing the crude protein fraction of the fruit body using a shotgun proteomics approach using multidimensional protein identification technology (MudPIT) by LC-MS/MS in an LTQ mass spectrometer [4]. Their protocol involved biphasic column separation prepared with reversed-phase resins followed by strong cation exchange material. The relative quantification of the identified proteins revealed several protein signatures that are highly abundant in the fruiting body. Functional classifications of the identified proteins were also provided by bioinformatics tools.

The manuscript produced by Stryinski et al., “Proteomic insights into the biology of the most important foodborne parasites in Europe”, provides an excellent overview of the applications of proteomic methods in studies on foodborne parasites and their potential use in targeted diagnostics [5]. Discovery proteomics methods were described for the characterization and selection of protein biomarkers for selected foodborne parasites such as waterborne parasitic species (e.g., *Cryptosporidium* spp., *Giardia lamblia*, *Entamoeba histolytica*, etc.), soil- and plant-borne parasitic species (e.g., *Echinococcus multilocularis*, *Toxocara* spp., *Ascaris* spp., *Fasciola* spp., *Trypanosoma cruzi*, etc.), meat-borne parasitic species (i.e., *Toxoplasma gondii*, *Trichinella* spp., *Taenia* spp., and *Sarcocystis* spp.) and seafood-borne parasitic species (i.e., Opisthorchiidae, *Angiostrongylus cantonensis*, *Diphyllobothrium* spp., *Paragonimus* spp., and Heterophyidae). Targeted proteomics methods were also described to search for peptide biomarkers selected in the discovery phase with high precision, sensitivity and reproducibility. This paper was mainly focused on the description of targeted proteomic methods proposed for Anisakidae detection in food products.

Finally, the article published by Carrera et al., “Proteomic strategies to evaluate the impact of farming conditions on food quality and safety in aquaculture products”, presents different proteomic strategies (discovery and targeted proteomics) to evaluate the impact of farming conditions on food quality and safety in aquaculture products [6]. Food quality, dietary management, fish welfare, stress response, food safety (biotic and abiotic hazards) and antibiotic resistance are the main proteomic techniques and strategies that are successfully covered in this review. The authors conclude by outlining future directions and potential perspectives, as the development and practical implementation of new advances based on protein microfluidics, protein biosensors and device digitalization offer promising research areas for the aquaculture industry and food authorities.

The “Proteomics and Food Analysis: Principles, Techniques, and Applications” Special Issue is an ideal and timely guide for researchers seeking to understand the proteome of any food biological sample. Finally, the Guest Editor, Dr. Carrera, wishes to express her gratitude to all the authors for their contribution in the preparation of this Special Issue.

## Data Availability

No applicable.
